# Role of Tranexamic Acid in Palliative Control of Bleeding in End-Stage Head and Neck Cancer: A Case Report

**DOI:** 10.7759/cureus.45534

**Published:** 2023-09-19

**Authors:** Vanshika Tripathi, Oshin Rai, Natalie Shaykh, Falguni Patel, Pramod Reddy

**Affiliations:** 1 Internal Medicine, University of Florida College of Medicine – Jacksonville, Jacksonville, USA

**Keywords:** oral tranexamic acid, anti-fibrinolytic therapy, palliative care, cancer of the head and neck, hemoptysis

## Abstract

Tumor-related bleeding is a common manifestation of end-stage head and neck cancer, and it can have a significant impact on a patient's quality of life. Tranexamic acid is an anti-fibrinolytic agent that has been shown to effectively control bleeding and reduce the need for transfusions in various hemorrhagic conditions. Here, we present the case of a patient with end-stage head and neck cancer experiencing recurrent episodes of bleeding, who was able to successfully achieve hemostasis after being treated with tranexamic acid. This case report highlights the role of tranexamic acid as a palliation agent that can help control the unpleasant bleeding symptoms of end-stage head and neck cancer and provide a better quality of life for patients.

## Introduction

Tranexamic acid is an antifibrinolytic medication that works by preventing the breakdown of blood clots. Its structure is similar to that of the amino acid lysine, and it works by competitively blocking the lysine-binding sites of plasminogen, subsequently preventing its conversion to plasmin. Plasmin is one of the key molecules that breaks down fibrin clots. By preventing plasmin formation, tranexamic acid stops fibrin from being broken down thereby preserving clot stability and promoting hemostasis [1-3]. Traditionally, tranexamic acid has been used to control bleeding in a wide variety of situations including surgeries, trauma, heavy menstrual bleeding, postpartum hemorrhage, hematologic disorders, and epistaxis [4-6]. Here, we present a case report demonstrating the effectiveness of tranexamic acid as a potential palliation agent used to alleviate bleeding in end-stage head and neck cancer.

## Case presentation

**Figure 1:**
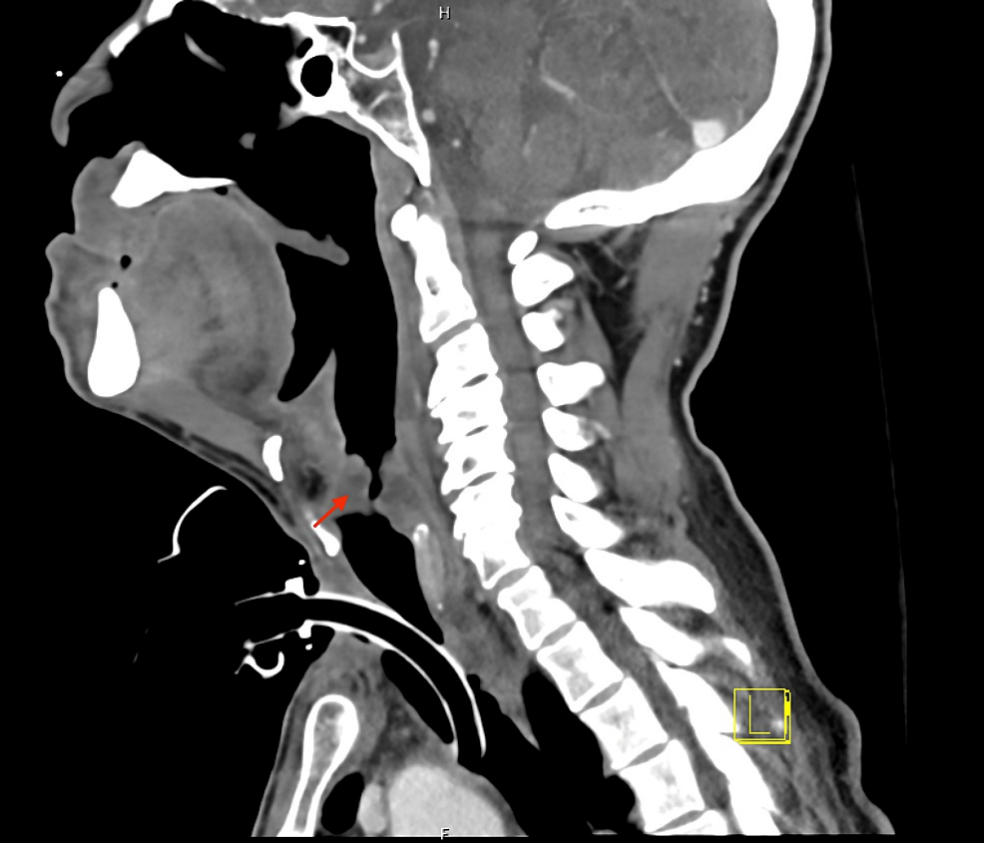
Neck (soft tissue) CT with IV contrast demonstrating the transglottic laryngeal mass (sagittal view)

**Figure 2:**
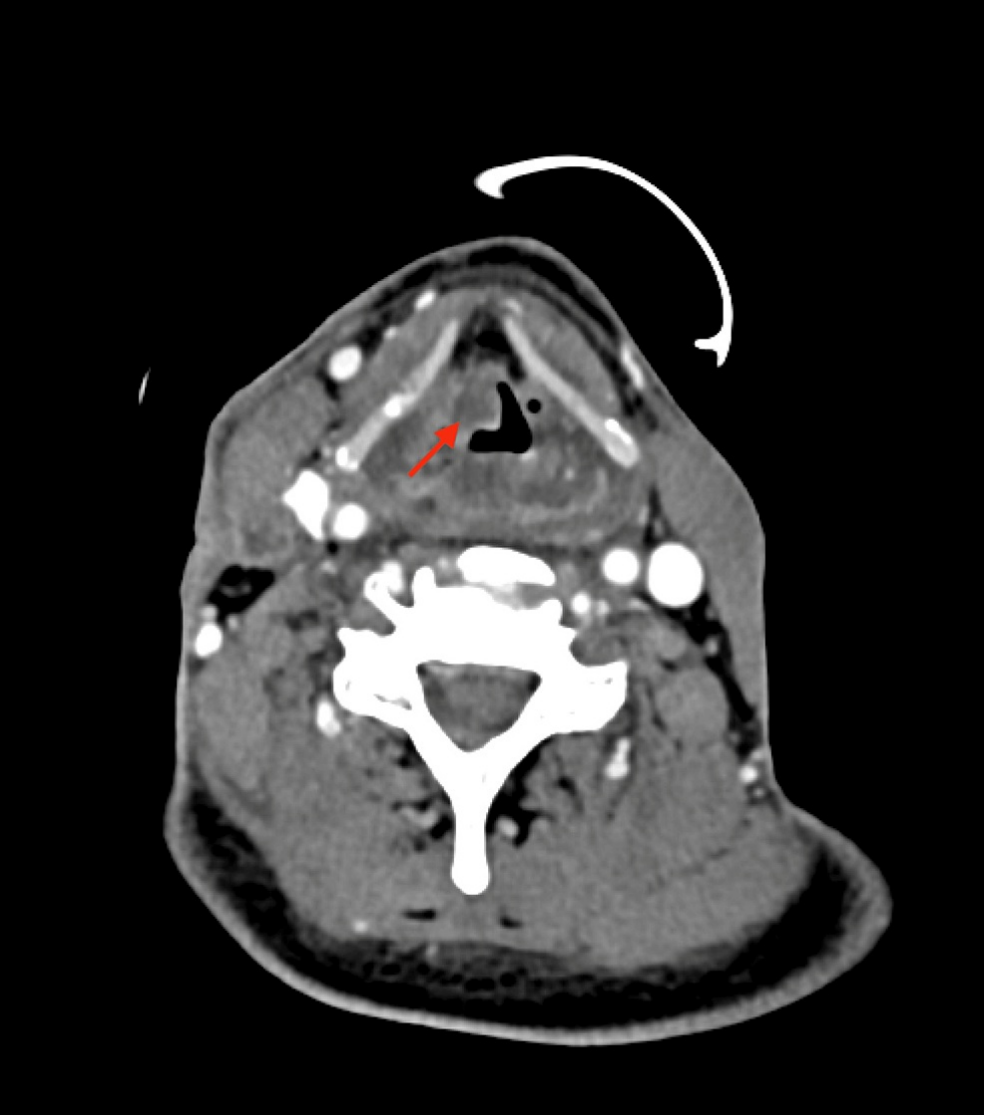
Neck (soft tissue) CT with IV contrast demonstrating the transglottic laryngeal mass (axial view)

**Figure 3:**
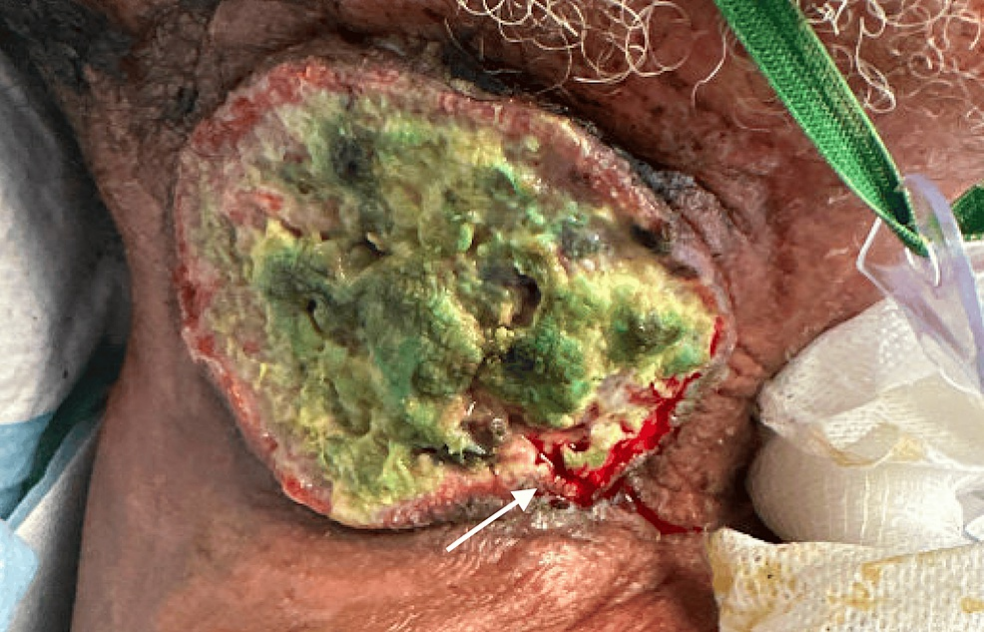
Fungating right neck mass with active bleeding

A 62-year-old male with locally advanced squamous cell cancer of the larynx status post chemoradiation therapy was initially brought into the ED due to acute altered mental status. This patient had previously undergone extensive treatment with multiple rounds of cisplatin chemotherapy and radiation, but had ultimately rejected a laryngectomy and developed significant disease progression including significant dysphagia and airway involvement that led to eventual tracheostomy and percutaneous endoscopic gastrostomy (PEG) tube placement (Figures 1-2). He had a prolonged hospitalization course during which his altered mental status improved with treatment of his methicillin-susceptible *Staphylococcus aureus* (MSSA) pneumonia and underlying psychiatric illnesses. Despite the resolution of his presenting complaint, this patient’s hospitalization was complicated by recurrent bouts of bleeding from a fungating right neck mass along with development of blood-tinged sputum from his tracheostomy site (Figure 3).

The recurrent bleeding from the patient’s neck mass impacted his ability to eat and speak, significantly affecting his quality of life. One night during this patient’s hospitalization, he developed severe bleeding from the tumor site requiring pressure to be held and transfusion of 1 unit of packed red blood cells. He achieved temporary hemostasis after being given a nebulized tranexamic acid treatment along with desmopressin, and did not have further bleeding events for the next 24 hours. Unfortunately, the next day he started to develop a recurrence of bloody sputum along with bleeding from his neck mass. The decision was then made to initiate high-dose oral tranexamic acid at 1,300 mg three times per day [7]. Since the initiation of the oral therapy, the patient did not have another bleeding occurrence and his hemoglobin remained stable for the remainder of hospitalization without requiring any further transfusions. He was able to enjoy pureed foods along with his tube feeds, and had an improvement in communication. Given that long-term hemostasis was achieved with the oral tranexamic acid, the patient was indefinitely placed on this regimen. He now awaits skilled-nursing facility placement without any further episodes of bleeding noted during his current inpatient hospitalization.

## Discussion

Tranexamic acid is an antifibrinolytic agent that is used acutely to control bleeding in various hemorrhagic settings. It comes in inhaled, oral, and intravenous forms, and is regularly used to achieve hemostasis in bleeding occurring during surgeries, trauma, epistaxis, hemoptysis, obstetric complications, and heavy menstruation. It works by preventing the activation of plasminogen to plasmin, and maintains pre-existing clot integrity by preventing fibrin breakdown. It is very rarely used as a chronic medication as its utility is more in acutely achieving hemostasis [1-3]. Our case highlights the potential role of long-term tranexamic acid use as a form of palliative care in advanced head and neck cancer. Despite optimizing wound care and local hemostatic treatments, our 62-year-old patient with advanced head and neck cancer had his quality of life impacted by the distressing manifestation of recurrent bleeding from his tracheostomy site and fungating neck mass. Considering his poor prognosis and frequent symptoms, the decision to continue him on long-term oral tranexamic acid therapy was made. He successfully achieved hemostasis without recurrence of bleeding during his current hospitalization for over three months since the initiation of tranexamic acid.

## Conclusions

Advanced head and neck cancer can be complicated by recurrent bleeding that can heavily impact the remaining lifespan of a patient already dealing with a grave prognosis. Our case report emphasizes how long-term tranexamic acid therapy seems to be a promising option for reducing bleeding episodes in patients with advanced head and neck cancers, thereby improving their quality of life. Further areas to explore through research include a comparison between oral, inhaled, and intravenous forms of tranexamic acid and whether they are equally efficacious as a palliative care therapy. Additionally, the utility of tranexamic acid as a palliative care hemostatic agent in other forms of malignancy that are complicated by bleeding needs to be studied.
